# Health-related quality of life experiences in children with bladder exstrophy-epispadias complex: a Swedish focus group study

**DOI:** 10.1007/s11136-026-04316-7

**Published:** 2026-06-19

**Authors:** Ulrika Svenninghed, Elin Öst, Gundela Holmdahl, Lisa Örtqvist, Magdalena Boije, Sofia Sjöström, Michaela Dellenmark- Blom

**Affiliations:** 1 Department of Pediatrics, Institute of Clinical Sciences, Sahlgrenska Academy, University of Gothenburg, Gothenburg, Sweden; 2 Department of Pediatric Surgery, the Queen Silvia Children’s Hospital, Sahlgrenska University Hospital, Gothenburg, Sweden; 3 Department of Pediatric Surgery, Astrid Lindgren’s Children’s Hospital, Karolinska University Hospital, Stockholm, Sweden; 4 Department of Women’s and Children’s Health, Karolinska Institutet, Stockholm, Sweden

**Keywords:** Bladder exstrophy, Epispadias, Health-related quality of life, Child, Rare disease, Focus group

## Abstract

**Purpose:**

Bladder exstrophy-epispadias complex (BEEC) is a rare urogenital malformation. Children risk long-term urinary and genital dysfunction. The study aimed to describe health-related quality of life (HRQoL) experiences among children with BEEC and ultimately guide the development of a BEEC-specific HRQoL questionnaire.

**Methods:**

Ten focus groups stratified by child age and sex were conducted in Sweden with 15 children aged 8–18, and 23 parents of children aged 2–17. Sessions were moderated, audio-recorded and transcribed verbatim. Reports of children’s BEEC-related HRQoL were extracted and content analyzed into categories sharing a common feature.

**Results:**

1713 statements of the child’s HRQoL (1076 from parents, 637 from children) were identified and grouped into seven HRQoL categories. *Somatic experiences/Physical consequences due to BEEC* reflected experiences of urgency, leakage, smell, infections, pain/discomfort and bodily variations. *Living with the choice of whether or not to be open* described children’s decision-making about revealing BEEC to others. *Social relationships* highlighted the importance of peers and articulated social vulnerability. *Adaptational needs due to bladder dysfunction* included special needs in relation to the night-time, time schedules and clothing. *Functioning in environments outside home* included experiences with public toilets, school and leisure activities. *Psychological impact* encompassed emotional consequences and perceptions of different appearance. *Growing up with BEEC* included thoughts about sexuality, parenthood, the future and independence.

**Conclusion:**

This first-reported focus group study in children with BEEC reveals physical, psychological, and social impacts across life domains. These findings justify and enable development of a disease-specific HRQoL questionnaire for children with BEEC.

**Supplementary Information:**

The online version contains supplementary material available at https://doi.org/10.1007/s11136-026-04316-7.

## Introduction

Bladder exstrophy-epispadias complex (BEEC) is a rare urogenital malformation with varying severity. The mildest form is isolated epispadias (E), where the bladder is intact, but the urethra is open, and the urinary sphincter in most cases is insufficient. In bladder exstrophy (BE), there is a closure defect of the lower abdominal wall. The pelvic floor muscles are underdeveloped, and the pelvic ring is open at the front. The bladder and urethra are open and exposed below a low-set umbilicus. Girls have a split clitoris with a narrow, short vagina, while boys have a short, wide and upward-curved penis. The most severe variant is one in which BE is combined with abnormalities of the intestinal tract and sometimes spinal cord, known as cloacal exstrophy (CE). BEEC is more common in boys [1, 2]. Its etiology is not completely understood, but likely attributable to genetic [3] and environmental factorsp [1].

Children with BEEC undergo multiple surgical procedures, starting in infancy. The main goal of surgical treatment is to achieve continence with preserved renal function, as well as cosmetically and functionally acceptable genitalia. Several surgical strategies are currently applied. While complete primary repair encompasses a single correction of the bladder, urethra and external genitalia, a modern staged repair spreads bladder closure, epispadia repair, and bladder neck reconstruction over three surgical interventions in boys, and two in girls. An adjunctive osteotomy and closure of the symphysis may be performed in order to reduce the likelihood of abdominal wall failure and bladder closure [1, 4, 5]. Urinary diversion, which reroutes and drains urine from its normal pathway, may be indicated when primary bladder closure or continence surgery fails, or where the upper urinary tract is at risk [1]. Both girls and boys may require additional reconstructive surgery during childhood. Procedures such as bladder augmentation with a catheterizable channel, such as Mitrofanoff, is warranted in many cases due to limited native bladder capacity [1, 6].

Up to 70% of children with BEEC suffer from urinary incontinence [7]. Additional bladder surgery is needed in up to 89% of the patients by the age of 18[8] and about 70% are in need of clean intermittent catheterization (CIC) for bladder-emptying [7, 8]. Health-related quality of life (HRQoL) refers to a multidimensional concept that represents the patient’s overall perception of the impact of disease and its treatment on physical, psychological, and social aspects of life, and is considered to be a core patient-reported outcome [9]. According to three recent reviews [10–12], generic HRQoL in patients with BEEC may be reduced compared to the general population [10], with a greater risk of emotional and behavioral problems during adolescence [10, 12]. Children with BEEC may have urinary tract infections (UTIs), scarring from multiple surgeries, and altered genital appearance/function, which also impacts on their HRQoL [10]. In a systematic review, Dellenmark-Blom et al. observed that previous studies assessed HRQoL heterogeneously, and no disease-specific HRQoL questionnaire was available [10]. However, a questionnaire of this kind is warranted to standardize an outcome that measures HRQoL aspects relevant to children with BEEC [13, 14]. Knowledge generated by implementing such an HRQoL questionnaire among children can inform clinical decision-making, healthcare delivery, educational services, patient support groups and policymakers [13, 15]. Qualitative research, particularly focus groups (FGs), in rare paediatric diseases has been shown to be imperative to capture patient and parent experiences for use in the development of a disease-specific HRQoL questionnaire [16–21]. Compared with individual interviews, FGs can provide children with a supportive peer environment in which they are recognized as experts of their lives, which may reduce the perceived power imbalance between the researcher and the participant. Interaction with peers can also encourage participants to share their experiences when they recognize commonalities with others, while also being comfortable to express both similarities and differences in their perspectives. A FG can also reduce pressure on individuals to respond to every question. In the context of a rare condition, families may not have previously met others living with the same diagnosis and may therefore experience a sense of isolation or feel that their experiences are unique. FGs can help mitigate this by providing an opportunity to connect with others who share similar circumstances [21–23] However, qualitative studies in children with BEEC are rare [24], and no FG study has been published. In addressing this gap, the primary study aim was to describe HRQoL experiences among children with BEEC using FG methodology. The long-term goal was to use this concept elicitation to guide the development of a disease-specific HRQoL questionnaire for children with BEEC according to current standards of PROM [25].

## Method

This study followed Standards for Reporting Qualitative Research [26] (Supplemental Material 1).

### Ethics

The study was approved by the Regional Ethical Review Board in Sweden on 15 March 2023 (Reference: 2021–06762-01). All children received age-adapted oral and written research information, which was approved by an external body, the Swedish Ethical Review Committee. The written research information to children was adapted to the age groups 8–11, 12–14 and 15–18 years. This information included a description that participation was voluntary, that children had the right to refuse their parents’ participation and that they could withdraw their participation at any point without a need to explain why. All children needed to give oral consent. All legal guardians and adolescents from age 15 years provided written informed consent.

### Study design

To provide a supportive and secure peer environment that recognizes children and parents as experts, and enable the collection of rich and in-depth data on HRQoL experiences related to BEEC, a FG study in children with BEEC and their parents was designed[27, 28] using deductive-inductive analysis approach. HRQoL was defined as the individual´s experience of how disease and treatment affect functioning, disability and perceived physical, psychological and social well-being[13, 29, 30]. In HRQoL research, pediatric patients are considered as the primary source of information [27]and children can usually self-rate their HRQoL reliably from the age of 8 years [31]. Parents can provide complementary information of their child’s HRQoL but may also be the only reporter when children cannot self-report due to young age or cognitive dysfunction [27]. This was taken into account also in FG design. A collaboration with the Swedish patient support group ILCO (Ileum Colon; Tarm-Uro-Stomi-Förbundet) was established. ILCOrepresenting families of children with BEEC participated in: designing the focus group study; developing the interview guide; managing ethical risks; and discussing research findings.

### Setting and study participants

In Sweden, 3–6 children with BEEC are born annually [32]. Considering this rarity, the goal was to recruit around 4 participants per FG. The inclusion criteria for study participation are presented in Table 1. To create group homogeneity in the FGs and a sense of security among the participants as recommended [27], the FGs were stratified based on child age (young children 2–7, school-age 8–12 and teenage 13–17 years) and sex (female/male). Hence, families were purposively sampled. Out of 75 families of children with BEEC aged 2–17 years who had received multidisciplinary follow-up care at two out of the four tertiary urological centres in Sweden, 39 families were invited to the FGs and 27 accepted the study invitation. One researcher, a urotherapist with a knowledge of BEEC and the project, contacted the families, provided verbal study information and sent the families written study information by post.

**Table 1:** Presentation of the inclusion criteria in the study

Children born with any of the following conditions: isolated epispadias, bladder exstrophy or cloacal exstrophy
Children participated from age 8 years One parent of each child from the child age range of 2–17 years participated
Received care at the Queen Silvia Children’s Hospital in Gothenburg, Sweden, or the Astrid Lindgren Children’s Hospital, Karolinska University Hospital, Stockholm, Sweden
Legal guardians and children aged ≥ 15 years sign written informed consent for study participation
To be able and fully understand the research information, give informed consent according to Swedish regulations/laws and to be able and participate in focus groups Parents of children and adolescents aged 15 years or older needed to be fluent in written and spoken Swedish Children needed to be fluent in spoken Swedish

### Data collection

In-person FGs were conducted in non-clinical facilities at the hospitals during 2022–2023 and were audio-recorded. FG discussions were facilitated by trained moderators with no prior clinical relationship to the participants, who ensured that all participants had an opportunity to contribute. There was one moderator at each study centre for children (US, EÖ), and three for parents (MDB, EÖ, LÖ), all of whom had a clinical health professional background. The moderator followed a semi-structured interview guide covering, among others, questions about HRQoL experiences(Supplemental material 2) developed based on our previous literature reviews on HRQoL in BEEC[10, 11], multidisciplinary clinical expertise and experiences from ILCO. A field assistant was present who made field notes of non-verbal cues, group interactions and compliance with the interview manual. The FG began with a moderator introduction to create a relaxed atmosphere, explain the procedure, and allow participants to become familiar with one another. The moderator started with a general question (e.g., *What is important in your life for you to feel well?*) and then gradually asked more specific questions to explore participants’ HRQoL experiences across different life domains (school, home, leisure), including specific aspects of BEEC. Follow-up and probing questions were used (e.g., *Would you like to tell us how?*, *How did it happen?*) while ensuring that all participants had the opportunity to contribute (e.g., *When you compare with your own experiences, do you agree or feel differently?*). At the end of the FG, the moderator offered the participants an opportunity to anonymously write down the experiences of BEEC that were important for them, but that they didn’t want to share openly within the group, and to put their notes directly into a locked “sealed box”, to be opened later only by researchers. Parents and their children participated at separate facilities located near each other. During the FGs, the children could interrupt the FG and visit their parent if needed.

### Data analysis

The FGs were transcribed verbatim by US (n = 1), MB (n = 2) and a professional company (n = 7). The FG transcripts and participants’ notes in the sealed box were analyzed using manifest qualitative content analysis [33], focusing on what the texts explicitly stated [33–35]. Examples of the procedure are illustrated in Supplemental material 3. One researcher (US) read the transcripts and used the HRQoL definition deductively to extract meaning units of HRQoL experiences in children with BEEC from them, then merged them line by line into Microsoft Excel 365 together with pseudonymized study participant information. US condensed the meaning units, continuously coded and sorted data inductively into subcategories/categories sharing a common feature. The transcripts were re-read by US to ensure that all content had been covered. A coding list with definitions and descriptions of the subcategories/categories was drawn up to facilitate a systematic identification pattern of content. A second senior researcher (MDB) continuously and independently reviewed the categorization process. The categorizations were discussed between US and MDB until consensus was obtained. Lastly, the categorizations were reviewed and agreed upon by all authors. Participant statements within each category were analyzed using descriptive statistics (n, %). Quotes from study participants were selected to demonstrate how transcript data aligned with identified categories.

### Methodological rigour

To ensure trustworthiness of the study, multiple criteria were addressed as shown in Table 2

**Table 2:** Presentation of concepts and their consideration in the present study to ensure methodological rigour

Aspect of methodological rigour	Definition of concept	How it was considered in the study
Triangulation	Use of multiple datasets, methods, and/or investigators to address a research question	Method triangulation: Two methods of data collection were used: focus groups and field notes Investigator triangulation: The research team included members with expertise in various areas, such as researchers, urologists, urotherapists, paediatric nurses and methodologists in quality-of-life research Theory triangulation: The interview guide was designed using several different theoretical and knowledge sources Data source triangulation: The study involved children and adolescents of different ages, as well as their parents, to capture multiple perspectives and validate the data
Credibility	Confidence in the truth of the data and how well the study captures participants’ experiences without omitting relevant insights	Interview template with open-ended questions, allowing participants’ own experiences to emerge rather than reflecting the interviewer’s preconceptions Follow-up questions were also utilized by the moderator to enable in-depth responses Multiple researchers engaged in the analysis to reduce bias Use of illustrative quotes from a broad range of participants to support interpretations
Dependability	Consistency and transparency of the research process; ability to track how data were coded and interpreted	Detailed documentation of the categorization process in Microsoft Excel Documenting each step of the analytic process, and application of a code-recode strategy throughout the analysis Version-tracking of Microsoft Excel files preserved throughout analysis To ensure consistency in the analysis and to ensure that no important categories were overlooked, a systematic coding process and coding list were employed Peer debriefings were held regularly in order to review coding decisions and to enhance consistency
Transferability	Extent to which findings may be applicable to other settings, populations or contexts	Purposeful stratification of participants’ characteristics, such as child age and child sex Description of the sample and healthcare setting provided Including the interview guide (supplementary material) used in the focus group interviews, which supports understanding of the context and methods
Confirmability	Objectivity and neutrality of the findings; the degree to which results are shaped by participants rather than researcher bias	Use of reflexivity and self-reflection Triangulation of data collection and analysis Moderators had no prior contact with the patient group before the study began The questions in the interview guide were designed to minimize bias To reduce the risk of interpretative bias, the analysis was confirmed within the research team
Data saturation	The point at which no new themes or insights emerge from the data; conceptual categories are comprehensive and well-grounded	Within focus groups: field assistant ensured all interview guide topics were covered, and all participants had the opportunity to contribute Across focus groups: saturation assessed by recurrence of categories across sessions

## Results

### Study participants

**Fig. 1:**
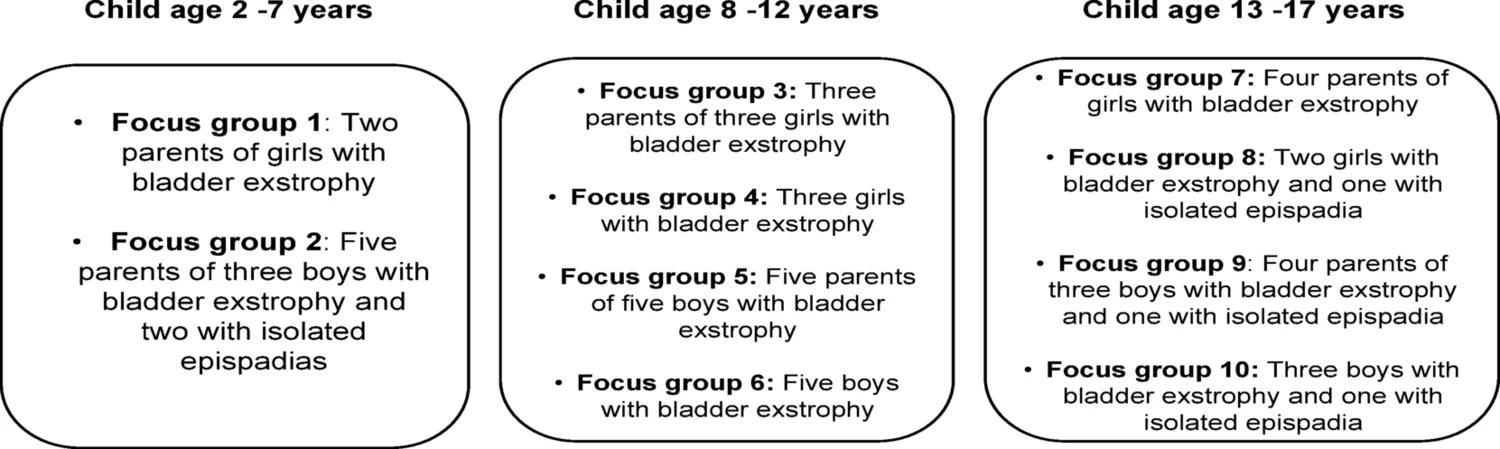
Presentation of the focus groups held with children who were born with bladder exstrophy or isolated epispadias and one of their parents stratified per age group and child sex

Out of 27 families, 23 families represented by 38 participants participated in ten FGs: 15 children with BEEC aged 8–17 years (four FGs), and 23 parents of children aged 2–17 years (six FGs). They lasted a total of 20 h and 4 min (FG duration range: 53 min to 2 h and 54 min). Children (median age 11 years) were born with BE (84%) or E (17%). The FGs, with the number of study participants and BEEC characteristics, are presented in Fig. 1, while a detailed overview of child and parent characteristics is given in Table 3.

**Table 3:** Characteristic of focus group participants

Child characteristics	
*Gender*	
Male	14(61%)
Female	9(39%)
*Condition and surgical treatment before the focus group*	
Bladder exstrophy	19/23(83%)
Early bladder closure (1–2 months)	19/19(100%)
Epispadias correction (12–18 months)	2/19(10%)
Epispadias correction before or after 12–18 months	9/19(47%)
Osteotomies	14/19(74%)
Continence-enhancing surgery (Mdn = 11 years old)	7/19(37%)
Bladder augmentation	5/19(26%)
Isolated Epispadias	4/23(17%)
Isolated Epispadia correction* (Mdn* = *1 years old)*	4/4(100%)
*Characteristics at time of the focus group*	
*Emptying of the bladder*	
Voiding spontaneously through urethra	13/23(57%)
CIC^a^ through the urethra	4/23(17%)
CIC^a^ via continent catheterizable channel e.g. Mitrofanoff	6/19(32%)
*Other physical impacts*	
Presence of an umbilicus	12/23(52%)
Presence of any difficulties walking	2/23(9%)
Pain or discomfort in the hips/pelvis	3/23(13%)
Presence of urinary leakage	18/23(78%)
The need to use incontinence care products	18/23(78%)
*Medication*	
Anticholinergic	2/23(9%)
Other	2/23(9%)

Parental information	
Mother	17/23(74%)
Cohabiting partner/married	19/23(83%)
National descent Sweden	20/23(87%)
Employment	21/23(91%)

^*a*^*CIC, Clean Intermittent Catheterization*

### HRQoL experiences

**Fig. 2:**
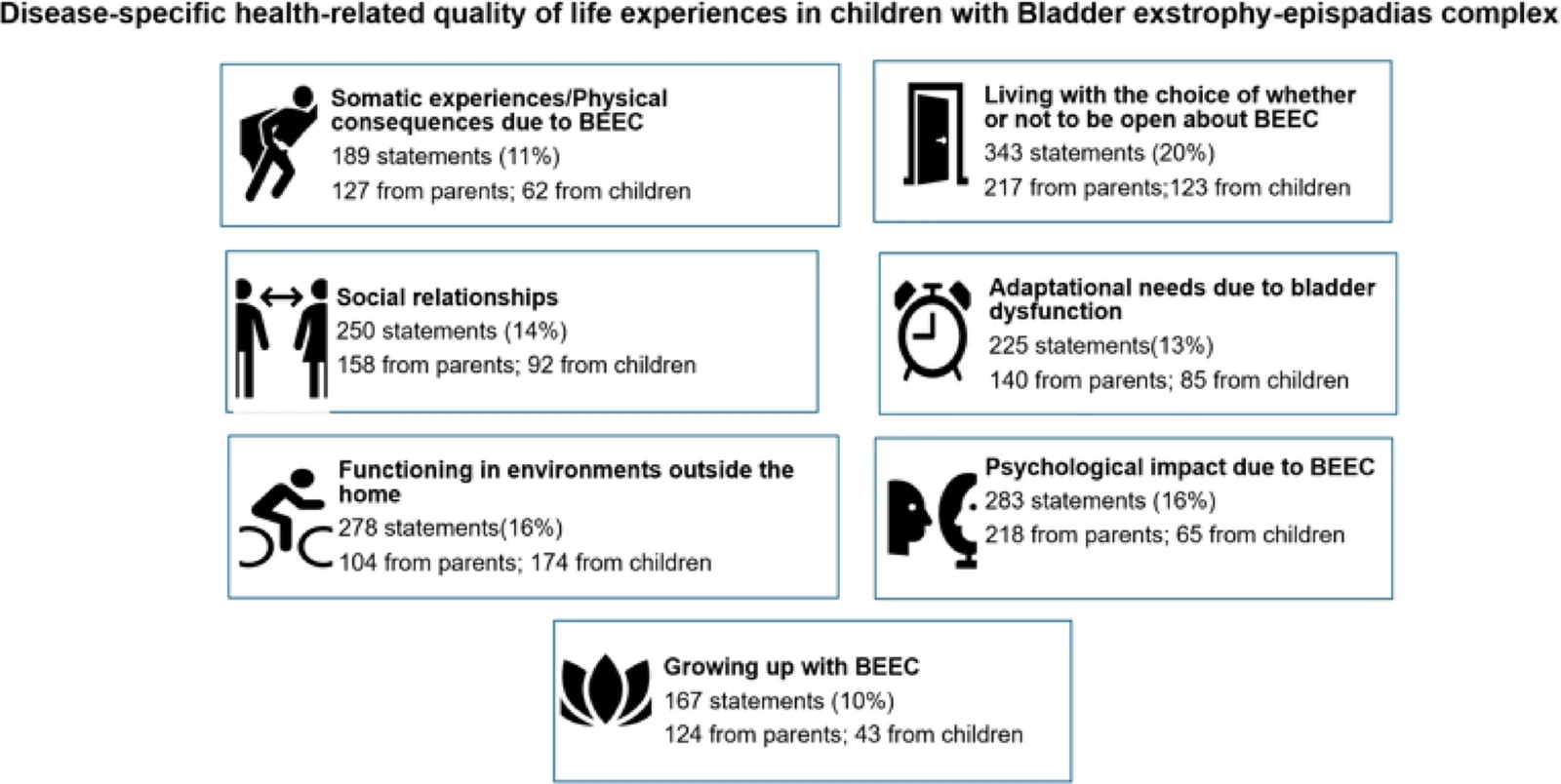
An overview of the categories generated from focus groups, with the total and specific number of statements of the child’s health-related quality of life reported by parents of children with bladder-exstrophy epispadias complex (BEEC) aged 2–17 years, and of children with BEEC aged 8–17 years, respectively

The content analysis yielded seven categories of HRQoL experiences in children with BEEC, including 1713 unique statements: 1076 made by parents of children with BEEC aged 2–17 years, and 637 made by children with BEEC aged 8–17 years. Data saturation was adequately achieved as judged by multiple researchers (Supplemental material 4). Figure 2 provides an overview of the categories, with the number of statements reported by parents and children with BEEC respectively, whereas Table 4 provides descriptives of their subcategories with illustrative quotes. An extended detailed version describing the category content is provided in Supplemental material 5. A summary of the categories follows below.

**Table 4:** Descriptives of categories, subcategories and examples of quotes from children with bladder exstrophy-epispadias and their parents

	Quotes
*Category 1. Somatic experiences/Physical consequences due to BEEC*	
*subcategories*	
1.1 Frequent and/or urgent urination	"You need to visit the toilet more often when you are born with BEEC, yes in the middle of class […], you have to " (Child aged 8–12 years with BEEC)
1.2 Urinary leakage	“My daughter is constantly leaking urine” (Father of a child 8–12 years with BEEC)
1.3 Smell of urine	“I've been able to micturate in the shower sometimes, in these situations you can feel the urine odour. I have a lot of soap in the shower and perfume and stuff.” (Child aged 8–12 years with BEEC)
1.4 Urinary infections	"You notice an infection by her fatigue, a small general feeling of illness which is sometimes so diffuse, so you've just sensed it […]. You send her off to school and then a couple of days later she collapses" (Mother of a child aged 12–17 years with BEEC)
1.5 Pain and discomfort	“And it [bladder] was so painful that she woke up from sleep, and at this point her quality of life was really poor” (Father of a child aged 8–12 years with BEEC)
1.6 Body and appearance	"She has big sister, she notices that they are different, the umbilicus and her looks below the waist, they do not look the same" (Mother of a child aged 2–7 years with BEEC)
*Category 2. Living with the choice of whether or not to be open about BEEC*	
2.1 The choice to tell or not to tell others about BEEC	”He [our son] doesn´t want anyone to know about it [BEEC] no friends, no one knows about BEEC” (Father of a child 8–12 years with BEEC)
2.2 Parents are a possible support to inform others about BEEC	“It´s probably mostly my parents who tell others about BEEC and my parents have told my little brother (Child aged 8–12 years born with BEEC)
2.3 Having someone to speak to in confidence about BEEC or not	“I mostly try to find a solution myself, and if I don’t find it, then I turn to my parents” (Child 12–17 years with BEEC)
2.4 The choice of showing or hiding parts of your body or supplies	” My son changes clothes in a toilet of the school’s changing room for sport class […]. He locks himself in the toilet”. (Father of a child 8–12 years with BEEC)
*Category 3. Social relationships*	
3.1 The importance of peer relationships	“[Important in life] is hanging out with friends” (Child aged 8–12 years with BEEC)
3.2 Receiving questions from others about BEEC	“Our son wants to be appreciated, but when he takes steps to socialize with others outside his usual friends and they start asking questions about BEEC, they are not welcome anymore” (Mother of a child 8–12 years with BEEC)
3.3 To be or feel socially excluded or exposed in vulnerable situations with other people	“It´s a bit annoying it´s just like that. She just stares at you” [in the bathhouse] (Child 8–12 years with BEEC)
3.4 About meeting others with BEEC	“They are four girls who are about the same age, who meet at camp [for children born with BEEC] and so on, and they mean quite a lot or very much to each other”(Mother of a child 8–12 years)
*Category 4. Adaptational needs due to bladder dysfunction*	
4.1 Time schedules to empty bladder	“You like to empty the bladder in time, […] then it gets a little stressful if you don't plan how to do it directly.” (Child 12–17 years with BEEC)
4.2 Nighttime	“I usually get up at night, I usually wake up because I need to visit the toilet”(Child aged 12–17 years with BEEC)
4.3 Clothes	“When we look at clothes, and regarding clothes for sport he has very distinct wishes that there should be zippered pockets, because then he can have incontinence pads with him in his pocket without them being visible to others” (Mother of a child aged 12–17 years with BEEC)
4.4 Dependent on bladder-emptying aids/incontinence products	“When I was younger, I had incontinence pads because it leaked all the time”. (Child aged 12–17 years with BEEC)
*Category 5. Functioning in environments outside the home*	
5.1 Public bathrooms or nature	“There is no building where my daughter can´t point out where all the toilets are” (Father of a child aged 8–12 years with BEEC)
5.2 Queues	“For example, it takes 40 min [in the Tivoli queue] then we have time to go through the queue and then when we just see Tivoli, then I say, I need to go to the toilet, and then we have to start standing in line again” (Child aged 8–12 years with BEEC)
5.3 To have or not to have a supportive school environment	“Our daughter has a agreement at school, that she has her own personal toilet, so it is not busy when she needs a toilet” (Father of a child aged 8–12 years with BEEC)
5.4 Participation in leisure activities	“My daughter has never slept over at someone else’s house because of BEEC”(Mother of a child aged 8–12 years)
*Category 6. Psychological impact due to BEEC*	
6.1 Perceived impact of having a different appearance/looks	"The last operation they did was to create a small umbilicus. I don't think it looks that great, but for her the thing was that she got an umbilicus. This was very important to her. She thought that with this, she was like everyone else" (Mother of a child 8–12 years with BEEC)
6.2 Emotional consequences of bladder dysfunction and repeated surgeries	“Leaking is probably the hardest thing with BEEC” (Mother of a child 8–12 years with BEEC)
6.3 Increased need for sense of security	“She needs to know what will happen, not just when we visit the hospital, but also in general, so that she can feel secure” (Mother of a child aged 8–12 years)
*Category 7. Growing up with BEEC*	
Future	"Yes, I have kind of thought about the future, if you are kind of pregnant, will the umbilicus to stand out? Do you do a caesarean section, or how do you do it?" (Child 12–17 years with BEEC)
7.2 Independence and Responsibility	“Our son who leaks urine, brings incontinence products with him. He makes sure it is always ready and always with him.” (Mother of a child 12–17 years with BEEC)
7.3 Intimacy and Sexuality/Sex	“When my son has a lot of questions about sex, I usually say, bring it up with dad or the surgeon, because it's a bit difficult” as a mom (Mother of child 8–12 years with BEEC)
7.4 When you get older and the body changes	"For me, I think, I kind of leaked less and less. Since I was in second grade, and then maybe in sixth grade or seventh grade, I quit using incontinence products" (Child 12–17 years with BEEC)
7.5 Changes after continence surgery	“There is usually a lot of snow in the winter. Well, that's a huge advantage because then you can manage clean intermittent catheterization trough Mitrofanoff to empty your bladder, you don't have to bring down your clothes all the way. It's so cold, but you can just pull up the zipper a little and you'll be fine. You don't get cold" (Child 12–17 years with BEEC)

BEEC, Bladder exstrophy-epispadias complex; CIC, Clean Intermittent Cathetherization; FG, focus group; HRQOL, health-related quality of life

### Somatic experiences/physical consequences due to BEEC

Many children experienced urgency and a need to find a toilet immediately. The children had different experiences of urinary leakage; some leaked intermittently, some occasionally, some constantly and uncontrollably, and others did not leak. They reported situations of urine odour, with a need to cover this up by using perfume or soap. Furthermore, they experienced recurrent UTIs, causing feelings of general illness, and sometimes leading to repeated treatment with antibiotics and school absence. Bladder pain was described when the bladder was full, in the morning, or during UTIs. Some boys reported penile pain. Discomfort was also reported during vesicostomy button use. Several children described awareness of having a different body appearance (hips, genitalia, scars) compared to peers.

### Living with the choice of whether or not to be open about BEEC

Children with BEEC carried with them a decision-making process: whether to tell others about BEEC or an operation due to BEEC, or whether to reveal the true reasons for any lengthy school absence. Some children with BEEC found it easy to open up; a few told their whole class, while others felt it was difficult to speak about it and only told closest friends or kept it to themselves. Children who explained BEEC to other people used few and simple words, whereas some children avoided the word “BEEC”. Children often had help from parents with informing teachers, classmates or relatives, but some children did not allow them to inform others. Most children trusted only their parents when it came to experiences related to BEEC. The children and parents also described the child’s need to hide their body and/or bladder-emptying aids from others. Several parents reported that their sons were particularly careful to conceal their genitals in changing rooms and public showers. Some children confessed that they did not want to be naked in front of others. Some children who were concerned about being seen with bladder-emptying aids /incontinence products carefully hid discarded in the bin, but this was of less concern at home.

### Social relationships

Having good friends was described as the most important element in the child’s wellbeing, someone who guarded the toilet during their visit, waited during toilet visits, and provided secure companionship. Many children with BEEC were asked questions about BEEC by their extended family, classmates and friends. If these questions were too intimate, they felt uncomfortable. Children with BEEC and their parents elaborated on vulnerability of social exposure. Children feared peers at school might work out the secret of their condition and discover incontinence products, giving them a reason to become socially excluded and bullied. They might also experience “other people” staring at them and make comments because of their BEEC. Children with BEEC had varying degrees of need to meet others with the same condition. Some children attended camps organized by the patient support group, which could foster shared experiences and long friendships. Meeting an adult with BEEC was highlighted as important to HRQoL.

### Adaptational needs due to bladder dysfunction

Children with BEEC needed to comply with time schedules in order to empty their bladder, using CIC or regular toilet visits. This meant that they needed to plan their day, which could be stressful. They needed to decrease fluid intake in the evening to avoid excessive urine production at night, remind themselves to urinate before bedtime, and straightaway in the morning, as well as to use incontinence protection/sheet protection due to night-time urine leakage. Some children relied on reminders, such as watches or parents. The children’s clothing was adapted—for example, wearing trousers with pockets in which to keep continence aids. Children needed to pack extra clothes for school or other activities. Parents often bought them several pairs of the same trousers in order to conceal the child’s need to change clothes after urinary leakage. The need for incontinence pads (of varying sizes) and catheters were central to everyday life, both practically and for feelings of security.

### Functioning in environments outside the home

Some children with BEEC needed toilets immediately, leaving no time to search for them. Parents emphasized how important it was for the child to know the locations of public toilets in advance, which boosted their confidence when leaving home. Trips required careful planning—identifying the locations of public toilets and disposing of incontinence supplies. Some children did not leave home out of fear they could not find a toilet in time. For this reason, train or plane travel was particularly challenging. Some children had positive experiences of using Mitrofanoff outside the home. Queues could be problematic for the children; disability cards, usually shown by parents, gave priority, though not all children were aware of this option. Some children felt a need to avoid such queues. A supportive school environment was important for their children's well-being—having a toilet with privacy, or lockers for storing incontinence supplies, pads and catheters. However, some children said that they kept their incontinence supplies in their backpacks at school, or they kept them in their own pockets. The relationship with the teacher was central. The teachers knew when the children urgently needed to visit the toilet, provided reminders for toilet visits and protected their privacy in the locker room before or after sport class. Several children with BEEC were supported by their parents in participating in leisure activities, due to their desire that BEEC should not be a barrier. The children who took part in sports had a need to manage their bladder before, during and after training. Their experiences of participating in sleepovers varied.

### Psychological impact due to BEEC

Children with BEEC expressed thoughts and feelings in response to bodily changes, particularly regarding the umbilicus, scars and genitalia. Some children felt uncomfortable with their appearance. They needed adult support in finding acceptance of BEEC. Children revealed that thoughts about “why you were born with BEEC”, and “what is it like to live with BEEC” were important to HRQoL. Urine leakage and constant toilet needs were said to be the two most difficult and limiting aspects for children with BEEC, potentially causing stress and fear. Their need for frequent access to a toilet made sleepovers at their friend’s home a concern. Some children felt tired of the need to frequently deal with UTIs, school absences and numerous medical appointments. Children expressed mixed emotions with regard to surgeries, including a fear of continence surgery and relief after continence surgery. Children with BEEC sought emotional security by means of good planning and a stable home environment. Many children, and particularly parents, also reported having a strong and close child-parent relationship. They both explained that when children felt emotionally secure, they had better self-esteem.

### Growing up with BEEC

Children with BEEC had thoughts, concerns and hopes regarding future; continence surgery, careers, the ability to travel and parenthood. As they grew, they took more responsibility for their condition. Parents of children with BEEC rarely discussed questions about intimate relationships or sexuality with their child, but believed their children did think about this in everyday life, and several children had tried to ask their parents directly. For boys, the ability to get an erection was important for HRQoL. Entering puberty, adolescents with BEEC underwent bodily changes, with some experiencing less urinary leakage and described as exhibiting typical teenage behaviours. Continence surgery led to significant improvements, with less urine leakage, reduced pain and fewer infections. It made following a strict schedule for bladder-emptying important, though use of Mitrofanoff was quick and practicable in different settings.

## Discussion

This is the first FG study of HRQoL experiences in children with BEEC, eliciting perceived physical, social and psychological impacts. Children with BEEC described somatic experiences including urgency, urine odour and pain, all of which are well known. Urinary incontinence is the most common experience in children with BEEC [7] and the most consistent risk factor for impaired HRQoL across all ages [10, 11]. Furthermore, children experienced bodily differences as compared to peers. Previous studies [36–38] have shown that 22–59% of young patients with BEEC experience impaired body perception due to genital appearance, abdominal scars or umbilicus differences supporting body perception items to form part of a BEEC-specific HRQoL questionnaire [27, 39] to ensure content validity.

Our study clearly highlighted a risk of social vulnerability among children with BEEC, meaning an increased risk of reduced social participation, functioning and well-being. Decision-making around openness regarding BEEC was a prominent topic in the FGs. These children’s fear of negative reactions from others has been described [24]. Furthermore, it has been shown that in > 25% of children, BEEC was hidden from peers, and in 16% from everyone outside the family [37]. In our study, many children with BEEC hid their assistive devices and avoided exposing their bodies, consistent with studies showing that 90% of males and 44% of females with BEEC avoid undressing or showering in a public changing room [37]. A reason behind not revealing BEEC to others may be attributable to concern about mistreatment [38]. As with other chronic conditions [40], children with BEEC also experienced being stared at or questioned by other people. In other studies, 12% of children with BEEC denied having close friendships [41], 59% reported an impact on peer relationships [37] and 68–100% had experienced bullying [36, 38]. In our study, families had varying experiences in terms of the degree of openness about BEEC. Teenaged girls with BEEC were difficult to recruit, and for those who voluntarily participated in the FGs, sharing experiences could still be challenging. Possible reasons are that adolescence and female gender generally are associated with an increase in mental health difficulties [42, 43]. Furthermore, some of the families had concealed BEEC up until the time of the FG. However, other families in our study were also fully open to others. In such cases parents often helped with communicating BEEC with schools and other adults. This is consistent with studies showing that 72% of BEEC individuals were able to communicate openly with parents or friends [36], and that adolescents with BEEC may even report friend relationships better than general norms [44]. As in Wilson et al. [24, 38], our study participants wished to have a person to speak to in confidence about their experiences, and in most cases, this meant parents. Children with BEEC valued security and closeness to their parents. A supportive and warm family environment is known to enhance mental health in children who face medical challenges [12, 45].

We also found that peer relationships were regarded as central to their HRQoL, in line with the Wilson et al. study [24]. Additionally, meeting peers with BEEC and participation in social contexts were deemed valuable for many. Many children with BEEC seemed to engage in leisure activities, emphasizing that BEEC should not limit them, which is consistent with previous findings [24, 46]. In earlier studies, sports were played to some extent by 65–92% [37, 41]. However, another study showed that 52% of individuals between 6 and 28 years with BEEC did not participate in parties, discos or movies [37]. In our study, experiences of camps and sleepovers varied. Those who underwent continence surgery described benefits for social participation, as supported by previous research [12].

In this study, the children’s daily lives were also influenced by day-time and/or night-time bladder management, revealing the focus, time and efforts invested by children in planning, routines and clothing—findings that were less detailed in previous research. It was critical for children with BEEC to know public toilet locations. However, children in our study with a Mitrofanoff channel, had positive experiences regarding the comfort and ease of emptying their bladders, even outside the home. A supportive school environment was critical for a good HRQoL in children with BEEC. This has likewise been said about their mental health [12]. However, school-related accommodations, such as separate toilets or special lockers, could become stigmatic, and therefore reinforce feelings of social exclusion [24].

The FGs revealed that sexuality and future parenthood were on children’s minds at a young age, though only some of them spoke in depth about this with their parents, which is new to the literature. Our FGs were stratified for child gender to make study participants comfortable, and FGs appropriate even for sensitive topics [47, 48]. Nevertheless, such issues can be difficult to discuss in a group setting and we therefore enabled sharing of experiences in the “sealed box”, where participants could share their experiences anonymously. Concerns about sexual relationships may be part of growing up with BEEC [24, 37, 45]. With regard to overall HRQoL, body image may worsen during their adolescence [10–12]. Since sexual dysfunction in adults with BEEC negatively impacts HRQoL [11], early tailored support around sexuality, intimacy, fertility and thoughts about future parenthood may be crucial in helping them to achieve a good HRQoL [49, 50].

The FG methodology allowed children, and their parents on their behalf, to express their views and needs [25, 27], consistent with the Convention on the Rights of the Child [51]. However, FGs may also pose ethical challenges [47]. Therefore, preparation focused on proactively minimizing risks and managing any emotional distress arising during or after the FGs. Measures included composing groups for homogeneity in age and sex, age-adapted research information, to potential study participants who were carefully informed that discussions were confidential and participation voluntary and were given designated time to reflect on their potential study participation.. The method provided the children with a safe peer environment and recognized them as experts in their own lives, while ensuring that their parents were close by, and offered a confidential option to submit written reflections for the researchers alone. Furthermore, we conducted follow-up phone calls two weeks post-session and pre-defined care pathways with multidisciplinary care, including with a pediatric urologist and psychologist, if researchers detected that any of the children were feeling somatically or mentally ill. This systematic approach not only ensured ethical safeguards for children and parents but also demonstrates a feasible and reproducible methodology for conducting FGs on sensitive topics, contributing to practices in qualitative research with vulnerable populations.

### Study strengths and limitations

This study is strengthened by careful preparational multidisciplinary work with ethical safeguards and collaboration with patient stakeholders, and contributes novel insights into HRQoL experiences in children with BEEC, and involved more participants than the only other reported qualitative study [24]. Our study achieved data saturation [52], despite varying and sometimes low participation rates in the individual FGs (Fig. 1, Supplemental material). Furthermore, the study complied with standards of methodological rigour [53]. Nevertheless, findings provide no indication of frequency of HRQoL problems, or the extent to which children with BEEC are affected. We did not analyze differences/similarities between children’s and parents’ experiences, but experiences reported by both were used as a source of information [21] in order to elicit HRQoL in children with BEEC. The study is also limited to the Swedish study setting with a centralized healthcare system, offering children with BEEC and their families multidisciplinary care with a urotherapist, urologist and psychologist as core team members throughout childhood. Surgical management of BEEC varies internationally. While moderators were trained, the quality of the FG data is dependent on moderator skills, and such skills may vary between several moderators [21]. Furthermore, in one FG, only two parents showed up as planned, limiting group dynamics.

## Conclusions

Children with BEEC have disease-specific HRQoL experiences in everyday life in relation to somatic experiences/physical consequences due to BEEC, living with a choice of openness about BEEC, impact on social relationships, adaptational needs due to bladder dysfunction, efforts to function in environments outside their home, a psychological impact due to BEEC, and experiences of growing up with BEEC. These findings underline a multidisciplinary care for children with BEEC that provides support in line with their needs. The study also shows that systematic preparation within a multidisciplinary team of clinicians, HRQoL researchers, and patient stakeholders can enable an ethically robust, feasible, and reproducible methodology for conducting focus groups in a rare disease context in a low-volume country, thereby contributing to best practices in qualitative research with vulnerable populations. In future research, these insights should generate items and so inform the development of a disease-specific HRQoL questionnaire for children with BEEC that is then subject to pilot- and field testing of its psychometric performance in Sweden and in other countries/languages, thus enabling a standardized PRO measure for this population. This instrument development also needs to address and evaluate HRQoL items in relation to child age- and sex. Future research of HRQoL experiences during the transition through young adulthood with BEEC is also warranted.

## Supplementary Information

Below is the link to the electronic supplementary material.

Supplementary Material 1

Supplementary Material 2

Supplementary Material 3

Supplementary Material 4

Supplementary Material 5

## Data Availability

The data that support the findings of this study are presented in the article and supplemental materials, and remaining pseudonymized data are available from the corresponding author upon reasonable request as long as it complies with the Ethical Approval and General Data Protection Regulation.
