# Supplementary material for: Health-related quality of life experiences in children with bladder exstrophy-epispadias complex: a Swedish focus group study
Source: Qual Life Res. 2026 Jun 19;35(8):208. doi: 10.1007/s11136-026-04316-7 (PMC13282288; doi:10.1007/s11136-026-04316-7)
Supplement: Supplementary file 1 — Supplementary Material 1 [file 11136_2026_4316_MOESM1_ESM.docx]

**Supplemental material 1 -** **Standards for Reporting Qualitative Research Checklist**

| No. | Topic | | Page |
| --- | --- | --- | --- |
| Title and abstract | | | |
| SI | | Title | 1 |
| S2 | | Abstract | 2 |
| Introduction | | | |
| S3 | | Problem formulation | 3-4 |
| S4 | | Purpose or research question | 3-4 |
| Methods | | | |
| S5 | | Qualitative approach and research paradigm | 4,6 |
| S6 | | Researcher characteristics and reflexivity | 6,17 |
| S7 | | Context | 4-6 |
| S8 | | Sampling strategy | 4-6 |
| S9 | | Ethical issues pertaining to human subjects | 4,25 |
| S10 | | Data collection methods | 5-6 |
| S11 | | Data collection instruments and technologies | 5-6 |
| S12 | | Units of study | 4-5 |
| S13 | | Data processing | 6-7 |
| S14 | | Data analysis | 6 |
| S15 | | Techniques to enhance trustworthiness | 7-8, Table 2 |
| Results/findings | | | |
| S16 | | Synthesis and interpretation | 8-15 |
| S17 | | Links to empirical data | Supplemental material 3 |
| Discussion | | | |
| S18 | | Integration with prior work, implications, transferability, and contribution(s) to the field | 17-18 |
| S19 | | Limitations | 18 |
| Other | | | |
| S20 | | Conflicts of interest | 25 |
| S21 | | Funding | 24 |
