# Supplementary material for: Health-related quality of life experiences in children with bladder exstrophy-epispadias complex: a Swedish focus group study
Source: Qual Life Res. 2026 Jun 19;35(8):208. doi: 10.1007/s11136-026-04316-7 (PMC13282288; doi:10.1007/s11136-026-04316-7)
Supplement: Supplementary file 2 — Supplementary Material 2 [file 11136_2026_4316_MOESM2_ESM.docx]

# Interview Guide in Focus Groups with children and adolescents born with bladder exstrophy-epispadias complex and their parents

# Moderator’s Introduction

• Thank the participants for coming.
• Introduce yourself (and the field assistant) by name.
• Explain why we are conducting this research, that there will be several different groups of children and parents, and that the same questions will be asked in all groups.
• Explain that today they will discuss different topics where **their perspectives are the most important.**

- If they don’t understand a question, they can say so and the moderator will explain further.
- Participation is voluntary.
- They are welcome to ask questions to each other.
- There are **no right or wrong answers**—everyone can think and feel as they wish.
- Everything is **anonymous**. No one outside the research team will know who said what. We, as researchers, will not share what is discussed in the focus group.
- (To children/teenagers): The only situation where something said might be shared outside the room is if we notice that you are not feeling well. In that case, we would need to tell your parent and help you. You are, however, free to talk with your parent about what was discussed in the focus group.

• Explain that the discussion will be audio **recorded**, but no one outside will know who said what. The purpose of the recording is so that we can remember and analyze everything that was said in all focus groups. The digital recorder will be placed openly on the table.

• Describe the **time frame**: the session will last a total of 1–2 hours, there will be a short break, and the timing of the break will be agreed upon together.

• For children aged 8–12 and 13–18 years: Remind them in which room their parent is. Tell them it is fine to stop the discussion at any time and to be accompanied to their parent by the field assistant if they wish.

# Main Questions for Parents/Guardians of Children Aged 2–18

When I [moderator] use the word *bladder exstrophy-epispadias*, I wonder if it is a term you use to describe your child’s condition. Or how do you usually refer to it in your family?
The moderator then ensures that all participants find the term understandable and acceptable and adjusts the wording if needed.

## Children’s Health-related Quality of Life and Coping

• What is important in your child’s life for them to feel well?
• Would you like to describe what it is like for your child to be born with bladder exstrophy-epispadias?
• Does bladder exstrophy-epispadias affect your child at school? If YES, would you like to describe how?
• Does it affect your child at home? If YES, how?
• Does it affect your child during leisure time? If YES, how?
• Are there areas of life that are **not affected** by your child being born with bladder exstrophy-epispadias? Which ones?
• If your children face difficulties/challenges because of the condition, how do they handle them? What helps them?
• How do you talk about the condition with your child?
• How do you talk about your child’s condition with others?
• What is difficult/what works well?

## Family and Parenthood

• Thinking back to when your child was born with bladder exstrophy-epispadias — what was that like for you as parents or expectant parents?
• How do you experience being a parent to a child with bladder exstrophy-epispadias today?
• Which areas of life are affected or unaffected?
• If this has been stressful for you as parents, how have you managed that stress?

## Experiences of Care and Treatment

• What experiences have you had with healthcare services?
• How do you experience your contact with healthcare services today?
• What is important for you to feel that you receive good care?

# Main Questions for Children Aged 8–12

When I [moderator] use the word *bladder exstrophy-epispadias*, I wonder if it is a word you use to describe your condition. Or what do you usually say?
The moderator ensures that everyone understands and feels comfortable with the term and adjusts wording if needed.

## Children’s Health-related Quality of Life and Coping

• What is important in your life for you to feel well?
• We want to understand more about what it is like for you being born with and having surgery for bladder exstrophy-epispadias.

- What is it like for you at school? Would you like to tell us how it is because of your condition?
- What is it like for you at home? Would you like to tell us how it is because of your condition?
- What is it like for you in your free time? Would you like to tell us how it is because of your condition?
  • Are there good and less good things in life because of bladder exstrophy-epispadias? Which ones?
  • If things are hard or difficult because of it, what do you usually do? What helps you?
  • Who do you usually talk to about your condition?
  • What is it like to talk with others about it? What is difficult/what works well?
  • What is it like to talk about it now in this group?

## Experiences of Care and Treatment

By healthcare, we mean the examinations, treatments, and appointments you might have with your doctor, nurse, or psychologist.
• Would you like to tell us what it’s like to take part in healthcare when you’re born with bladder exstrophy-epispadias?
• What do you think and feel about healthcare?
• What is important to make healthcare feel good for you?

# Main Questions for Teenagers Aged 13–18

When I [moderator] use the word *bladder exstrophy-epispadias*, I wonder if it is a term you use to describe your condition. Or what do you usually say?
The moderator ensures everyone understands and is comfortable with the term and adjusts wording if needed.

## Health-related Quality of Life and Coping

• What is important in your life for you to feel well?
• We want to understand more about what it’s like for you being born with and having surgery for bladder exstrophy-epispadias.

- Does bladder exstrophy-epispadias affect you at school? If YES, would you like to tell us how?
- Does it affect you at home? Would you like to tell us how?
- Does it affect you during your free time? If YES, would you like to tell us how?
  • Are there fun and less fun things in life because of bladder exstrophy-epispadias? Which ones?
  • If you face challenges or difficulties because of the condition, how do you handle them? Can you give examples (coping)?
  • Who do you usually talk to about your condition?
  • What is it like to talk with others about it? What is difficult/what works well?
  • What is it like to talk about it now in this group?

## Experiences of Care and Treatment

By healthcare, we mean the examinations, treatments, and appointments you might have with your doctor, nurse, or psychologist.
• Would you like to tell us what experiences you’ve had with healthcare when you’re born with bladder exstrophy-epispadias?
• What do you think and feel about healthcare today?
• What is important for you to make healthcare feel good?

# Example Follow-Up Questions for the Moderator

• Would you like to tell us about a situation where this happened?
• How did it happen?
• How did such a situation affect you?
• What was the hardest part?
• What was the easiest part?
• For the others: when you compare with your own experiences, do you agree or feel differently?

If something said isn’t clear, ask the participant to explain it in another way:
• “Can you say that in another way?” or “Can you tell us more about that?”
For children, especially the youngest, you can ask them to tell a story about a situation.

# Approx. 10–15-minute break with snacks.

# Part B – Writing/Drawing Personal Thoughts, Feelings, and Experiences

The second part of the focus group aims to give participants the opportunity to write down thoughts, feelings, and experiences they do not wish to share with the group.

## Suggested text for the moderator (example from the children’s group):

When we think about a questionnaire on the quality of life for children and young people born with bladder exstrophy-epispadias that we plan to create — what questions would *you* ask a child your age who was born with bladder exstrophy-epispadias? Anything you draw or write will **not be shown** to anyone else in this group. You can fold it and place it in this box [a sealed box that cannot be opened]. Only the researchers will look at it, to understand more. You may think about and share anything that comes to mind…

# Moderator’s Closing

Before the focus group ends, the moderator should summarize the topics and answers discussed during the session and give participants the opportunity to correct or add information. Then, explain how the information will be used.

Thank the children/teenagers/parents for their participation and end the session in a warm and friendly manner.
At the end, let the participants leave the room.
Turn off the recorder once everyone has left.
The moderator should remain for a short while after the focus group to receive any individual participant reflections or reactions.
