# Supplementary material for: Health-related quality of life experiences in children with bladder exstrophy-epispadias complex: a Swedish focus group study
Source: Qual Life Res. 2026 Jun 19;35(8):208. doi: 10.1007/s11136-026-04316-7 (PMC13282288; doi:10.1007/s11136-026-04316-7)
Supplement: Supplementary file 3 — Supplementary Material 3 [file 11136_2026_4316_MOESM3_ESM.docx]

| Supplemental material 3. Illustrative example of a manifest analysis procedure | | | | |
| --- | --- | --- | --- | --- |
| Meaning unit | Condensed meaning unit | Code | Sub-category | Category |
| *They do everything they can to [hide it], I mean, she comes home with bags of pads and catheters so she doesn't throw them in a paper bin where they can be seen, or throw them away at her friend's house because she sleeps over at friends' houses, but she would never do that if it could be seen, so she would never throw them away there, so you find them in the car, tucked under the seat and...* | *She does everything she can to hide assistive devices – don't leave them where anyone can see them, hide them in the car.* | *Hiding assistive devices and keeping them from others* | *The choice of showing or hiding parts of your body or supplies* | *Living with the choice of whether or not to be open about BEEC* |
| *If it takes about two hours, then I have to go to the toilet, but there isn't one where we do sports, so we play outside. There's a small spot [in the bush]—you can see through it, but if you bend down a little and stand by the bushes, you can pee there if you have a ‘rich thing’ with you."* | *No toilet available during sport training – solves this by clean intermittent catheterization behind the bushes* | How to empty the bladder during sports and leisure time | *Public bathroom or nature* | *Functioning in environments outside home* |
