# Supplementary material for: Health-related quality of life experiences in children with bladder exstrophy-epispadias complex: a Swedish focus group study
Source: Qual Life Res. 2026 Jun 19;35(8):208. doi: 10.1007/s11136-026-04316-7 (PMC13282288; doi:10.1007/s11136-026-04316-7)
Supplement: Supplementary file 4 — Supplementary Material 4 [file 11136_2026_4316_MOESM4_ESM.docx]

The ten FGs generated rich data from altogether 20 hours, 4 minutes (range 53 minutes-2 hours, 54 minutes). FGs were analyzed continuously by the first (US) and last author (MDB). Theoretical saturation was achieved across the focus group stratified for child age group 2-7, 8-12 and 13-17 years and child sex (male/females), as no additional categories emerged from the data collection, and all categories have been explored, identified, and completed. The category “Functioning in environments outside home” was not identified in the reports from parents of females aged 2-3 years old, but not expected in this child age. In comparison, the focus group with males aged 2-7 had a wide-spread age range. Therefore, data saturation was reached as judged by multiple researchers.

Furthermore, field notes confirmed all interview guide topics were covered and that all participants had the opportunity to contribute to the discussion. In six focus groups, it was noted that the atmosphere was first hesitant or tense among the participants. However, the participants became increasingly relaxed when someone started opening up creating recognition and generating thoughts among the other participants. All focus groups were described to reach an atmosphere described in terms of trust, participants showing interest, openness, sense of security and recognition into each other’s stories. In the FG with males aged 13-17 years, sensitive topics to discuss openly seemed to be related to puberty and emotional experiences. In one parent FG, scars and bullying seemed sensitive aspects to discuss openly. The FG’s duration of the open discussion varied. The shortest duration was 53 minutes and included teenage girls with bladder exstrophy-epispadias. In this teenage FG, one girl chose to interrupt her participation, but the other participants continued the discussion. Starting with brief answers on the moderator’s questions, they after a while opened up and asked each other questions, including about fertility and pregnancy. In the FGs, many expressed interests into staying in contact with peers.

All participants were offered to describe experiences anonymously in the secret box, and it was only used by children, not parents.

Data saturation was confirmed across the listed FGs below in the table and within the FGs.

| **Focus groups** | | **Somatic experiences/physical consequences due to BEEC** | **Living with the choice of whether or not to be open about BEEC** | **Social relationships/interactions related to BEEC** | **Adaptional needs due to bladder (dys)function** | **Functioning in environments outside the home** | **Psychological impact due to BEEC** | **Growing up with BEEC** |
| --- | --- | --- | --- | --- | --- | --- | --- | --- |
| **2-7** | **Boys (parents)** | - **Category identified** - **Field notes support saturation** | - **Category identified** - **Field notes support saturation** | - **Category identified** - **Field notes support saturation** | - **Category identified** - **Field notes support saturation** | - **Category identified in boys** - **Field notes support saturation** | - **Category identified** - **Field notes support saturation** | - **Category identified** - **Field notes support saturation** |
|  | **Females (parents)** |  |  |  |  |  |  |  |
| **8-12** | **Boys (parents)** | - **Category identified** - **Field notes support saturation** | - **Category identified** - **Field notes support saturation** | - **Category identified** - **Field notes support saturation** | - **Category identified** - **Field notes support saturation** | - **Category identified** - **Field notes support saturation** | - **Category identified** - **Field notes support saturation** | - **Category identified** - **Field notes support saturation** |
|  | **Females (parents)** |  |  |  |  |  |  |  |
|  | **Boys (children)** |  |  |  |  |  |  |  |
|  | **Females (children** |  |  |  |  |  |  |  |
| **13-17** | **Boys (parents)** | - **Category identified** - **Field notes support saturation** | - **Category identified** - **Field notes support saturation** | - **Category identified** - **Field notes support saturation** | - **Category identified** - **Field notes support saturation** | - **Category identified** - **Field notes support saturation** | - **Category identified** - **Field notes support saturation** | - **Category identified** - **Field notes support saturation** |
|  | **Females (parents)** |  |  |  |  |  |  |  |
|  | **Boys (children)** |  |  |  |  |  |  |  |
|  | **Females (children** |  |  |  |  |  |  |  |
