# Supplementary material for: Health-related quality of life experiences in children with bladder exstrophy-epispadias complex: a Swedish focus group study
Source: Qual Life Res. 2026 Jun 19;35(8):208. doi: 10.1007/s11136-026-04316-7 (PMC13282288; doi:10.1007/s11136-026-04316-7)
Supplement: Supplementary file 5 — Supplementary Material 5 [file 11136_2026_4316_MOESM5_ESM.docx]

| **Table 4. Descriptives of subcategories and examples of quotes from children with bladder exstrophy-epispadias and their parents** | | |
| --- | --- | --- |
| **1. Somatic experiences/Physical consequences due to BEEC** | | |
| **Subcategory** | **Content of subcategory** | **Quotes** |
| 1.1 Frequent and/or urgent urination | - Some children said that they could feel the need to micturate, while others could not - Some children described a feeling of urgency, meaning they needed to find a toilet immediately - Some children also associated carbonated drinks, such as soda, with increased frequency | "You need to visit the toilet more often when you are born with BEEC, yes in the middle of class […], you have to " (Child aged 8-12 years with BEEC) |
| 1.2 Urinary leakage | - Experience of urine leakage intermittently throughout their lives, some children leaked occasionally, some leaked constantly and uncontrollably | “My daughter is constantly leaking urine” (Father of a child 8-12 years with BEEC) |
| 1.3 Smell of urine | - Living with a risk of urine odour - Being bothered by urine odour - Some parents said that their children always had bacteria in their urine, which was a main reason of their smell - Attempts to hide urine smell by using perfume or soap | “I've been able to micturate in the shower sometimes, in these situations you can feel the urine odour. I have a lot of soap in the shower and perfume and stuff.” (Child aged 8-12 years with BEEC) |
| 1.4 Urinary infections | - Experience of recurrent urinary tract infections, not always accompanied with fever, but with a general feeling of illness, and repeated courses of antibiotics leading to school absence | "You notice an infection by her fatigue, a small general feeling of illness which is sometimes so diffuse, so you've just sensed it […]. You send her off to school and then a couple of days later she collapses" (Mother of a child aged 12-17 years with BEEC) |
| 1.5 Pain and discomfort | - Bladder pain in the morning or during urinary tract infections - Some boys reported experiences of penile pain - Experience of discomfort during use of vesicostomy button | “And it [bladder] was so painful that she woke up from sleep, and at this point her quality of life was really poor” (Father of a child aged 8-12 years with BEEC) |
| 1.6 Body and appearance | - Some children felt BEEC led to impact on body appearance; different appearance of the hip, genitalia, and the presence of scars compared to peers/siblings | "She has big sister, she notices that they are different, the umbilicus and her looks below the waist, they do not look the same" (Mother of a child aged 2-7 years with BEEC) |
| **2. Living with the choice of whether or not to be open about BEEC** | | |
| **Subcategory** | **Content of subcategory** | **Quotes** |
| 2.1 The choice to tell or not to tell others about BEEC | - There was a decision-making process; to tell or not to tell others about being born with BEEC, an operation due to BEEC and/or reasons for lengthy school absence - Some children felt it was easy to be open, some children that it was difficult - A few children told close friends about BEEC - Sometimes siblings were told about BEEC - If or when telling others about BEEC, the choice was to do it briefly and with simple words - The choice to tell also appeared when encountering questions from others - Some children explained only the practical things in the care related to BEEC, instead of mentioning “BEEC”, and some children never mentioned that the word “BEEC” | ”He [our son] doesn´t want anyone to know about it [BEEC] no friends, no one knows about BEEC” (Father of a child 8-12 years with BEEC) |
| 2.2 Parents are a possible support to inform others about BEEC | - Some children permit their parents to inform school, school-class or other people about BEEC, while some do not - Information to others about BEEC occurred at school meetings or situations when their children were not at home - Some parents also informed their child’s siblings about BEEC, whereas in other families it was kept a secret from them | “It´s probably mostly my parents who tell others about BEEC and my parents have told my little brother (Child aged 8-12 years born with BEEC) |
| 2.3 Having someone to speak to in confidence about BEEC or not | - Most of the children with BEEC only trusted their parents with situations they encountered in life or shared experiences of mental health problems related to BEEC - Children with BEEC knew about the possibility to speak with a professional about their experiences if needed, but no child described ever talking to a professional about BEEC | “I mostly try to find a solution myself, and if I don’t find it, then I turn to my parents” (Child 12-17 years with BEEC) |
| 2.4 The choice of showing or hiding parts of your body or supplies | - Children with BEEC experienced living with the choice to show or hide body parts, such as scars, genitalia and/or bladder-emptying aids/incontinence products from others - It was most difficult to show your genitalia - Several boys told they were careful to hide their penis in the locker room and in public shower rooms and changed clothes privately in the toilet - Some children experienced concern, but mostly not in the secure environment like their home | ” My son changes clothes in a toilet of the school’s changing room for sport class […]. He locks himself in the toilet”. (Father of a child 8-12 years with BEEC) |
| **3. Social relationships** | | |
| **Subcategory** | **Content of subcategory** | **Quotes** |
| 3.1 The importance of peer relationships | - Having friends was most important for well-being - A good friend can act as guardian while you are visiting the toilet and someone to play with securely without needing their parents close | “[Important in life] is hanging out with friends” (Child aged 8-12 years with BEEC) |
| 3.2 Receiving questions from others about BEEC | - Several children experienced questions about BEEC from the extended family and peers - It felt difficult for children to answer other people’s questions about BEEC - Several children didn’t want to spend time with peers who had too many questions | “Our son wants to be appreciated, but when he takes steps to socialize with others outside his usual friends and they start asking questions about BEEC, they are not welcome anymore” (Mother of a child 8-12 years with BEEC) |
| 3.3 To be or feel socially excluded or exposed in vulnerable situations with other people | - Other people make comments about a different look - Children felt worry or fear of social exclusion - Children felt that other people are staring at them because of BEEC - Children experienced peers who would try to break into the locker in school, where bladder-emptying aids/incontinence products were kept, ie risking their condition to become revealed - Some children encountered reactions from others; hesitancy to why they had a disability card | “It´s a bit annoying it´s just like that. She just stares at you” [in the bathhouse] (Child 8-12 years with BEEC) |
| 3.4 About meeting others with BEEC | - Camps organized by the patient association were important opportunities to meet other children born with BEEC - Some children felt that meeting others were valuable, and some children expressed a lack of interest in such meetings - For some children camps established lifelong friendships with opportunities to share experiences - Other children with BEEC were described to be unaware of why they visited the camp but had fun with the other children - Meeting an adult with BEEC was highlighted as important to HRQoL | “They are four girls who are about the same age, who meet at camp [for children born with BEEC] and so on, and they mean quite a lot or very much to each other”(Mother of a child 8-12 years ) |
| **4. Adaptational needs due to bladder dysfunction** | | |
| **Subcategory** | **Content of subcategory** | **Quotes** |
| 4.1 Time schedules to empty bladder | - Need to follow time schedules to empty their bladders, either the child used a intermittent clean catheterization via a Mitrofanoff channel to empty the bladder timely or needed to regularly go to the toilet to reduce urine leakage - Time tracking meant that children needed to plan their day, which could make them feel stressed - Need for reminders, such as watches or by other adults/their parents | “You like to empty the bladder in time, […] then it gets a little stressful if you don't plan how to do it directly.” (Child 12-17 years with BEEC) |
| 4.2 Nighttime | - Several children needed to decrease fluid intake in the evening to avoid excessive urine production at night, think carefully about urinating before bedtime and directly in the morning - Several children needed incontinence products and sheet protection due to nighttime urine leakage - Some children needed to set their clocks at night to allow for an additional toilet visit and some children received help by parents at night - For a few children, daytime tiredness was a concern | “I usually get up at night, I usually wake up because I need to visit the toilet”(Child aged 12-17 years with BEEC) |
| 4.3 Clothes | - Children needed to adapt clothes in which keep their bladder-emptying aids/incontinence products, for example by buying trousers with pockets and zippers within which continent aids were kept - Children needed to have several pairs of the same pants as it could hide the child’s need to change clothes after urinary leakage - Children needed to pack extra clothes for school or for other activities - Some children needed to use underwear’s with built-in incontinence protection | “When we look at clothes, and regarding clothes for sport he has very distinct wishes that there should be zippered pockets, because then he can have incontinence pads with him in his pocket without them being visible to others” (Mother of a child aged 12-17 years with BEEC) |
| 4.4 Dependent on bladder-emptying aids/incontinence products | - Children needed to use incontinence pads and catheters as central to managing everyday life, both practically and for security - Children showed need for using incontinence pads of various sizes to handle urine leakage, and for changing pads several times a day - Children had practical experience of CIC, such as bringing catheters in a toilet bag or backpack, having a disability card, and the need to pack necessary supplies | “When I was younger, I had incontinence pads because it leaked all the time”. (Child aged 12-17 years with BEEC) |
| **5. Functioning in environments outside the home** | | |
| **Subcategory** | **Content of subcategory** | **Quotes** |
| 5.1 Public bathrooms or nature | - For children it was important to know locations for public toilets in advance, as this boosted the children’s confidence and encouraged them to leave home - The children’s trips required careful planning, including identifying locations of the public toilets and knowledge of where to dispose of incontinence products. Some children avoided leaving home out of fear they could not find a toilet in time - Children who travelled by train or plane encountered particular challenges, necessitating careful planning of toilet visits before or after the travel - Some children also shared their positive experiences of using the CIC via Mitrofanoff channel in public, when there was no toilet available | “There is no building where my daughter can´t point out where all the toilets are” (Father of a child aged 8-12 years with BEEC) |
| 5.2 Queues | - Children with BEEC described problems waiting in a long queue for either toilet visits or visiting other public events, when having an urgent need to find a toilet. Children described attempts to avoid such queues because they were afraid of urinary leakage. - Some children used a disability card to get priority access toilets, preferred a parent to show the card for them, but not all children knew about this option | “For example, it takes 40 minutes [in the Tivoli queue] then we have time to go through the queue and then when we just see Tivoli, then I say, I need to go to the toilet, and then we have to start standing in line again” (Child aged 8-12 years with BEEC) |
| 5.3 To have or not to have a supportive school environment | - Having a supportive school environment and a good relationship with the teacher were important aspects of the children's wellbeing - In some cases, the school did not live up to support the child’s needs - Children needed school adaptation due to BEEC e.g. having a toilet with privacy, and some children preferred when the toilets at school were near the classroom and lockers for storing incontinence pads and catheter - Some children said that they kept their incontinence supplies in their backpacks at school rather than in the locker or kept them in their own pockets - Several children said that their teachers knew when they urgently needed to use the toilet and would let them go whenever necessary - Some teachers helped by reminding children when it was time to go to the toilet and supported those who needed more privacy when changing clothes before or after sport class | “Our daughter has a agreement at school, that she has her own personal toilet, so it is not busy when she needs a toilet” (Father of a child aged 8-12 years with BEEC) |
| 5.4 Participation in leisure activities | - Children and parents made efforts to enable participation in leisure activities such as sports, camps, and sleepovers - Several children received support from their parents in pursuing the activities they were interested in, emphasizing that BEEC should not be a barrier - Several children were involved in multiple sports and described the need to manage their bladder before, during, and after training - Some children had concern or chose to avoid them due to their condition - Participation in sleepovers varied. While some children attended without concern, others chose to avoid them due to their condition. - In some cases, the decision not to participate in leisure activities came from the child, while in others, it was the parents who advised against it | “My daughter has never slept over at someone else’s house because of BEEC”(Mother of a child aged 8-12 yeras) |
| **6. Psychological impact due to BEEC** | | |
| **Subcategory** | **Content of subcategory** | **Quotes** |
| 6.1 Perceived impact of having a different appearance/looks | - Thoughts and feelings about the bodily changes associated with BEEC, particularly regarding the umbilicus, scars and genitalia - Children sometimes felt uncomfortable with or disapproval of their appearance - Some children needed adult support to find acceptance and understanding, that it is not only BEEC that can make people different | "The last operation they did was to create a small umbilicus. I don't think it looks that great, but for her the thing was that she got an umbilicus. This was very important to her. She thought that with this, she was like everyone else" (Mother of a child 8-12 years with BEEC) |
| 6.2 Emotional consequences of bladder dysfunction and repeated surgeries | - Urinary leakage and the constant need to use the toilet were the two most difficult and limiting aspects of BEEC - The need for frequent access to a toilet made the children’s daily planning more challenging - Frustration and feeling tired of urinary tract infections, school absence due to illness and the numerous medical appointments - Children’s potential sleepovers at their friend’s home were common sources of concerns - Some children described fear of undergoing surgery again, while other children described feeling relief following continence surgery. - Some children were described to not need additional healthcare despite BEEC, not to be worried about scars or umbilicus, and not to experience a negative impact on their life due to BEEC. | “Leaking is probably the hardest thing with BEEC” (Mother of a child 8-12 years with BEEC) |
| 6.3 Increased need for sense of security | - Children with BEEC showed an increased need to find emotional security, and were dependent on a good planning and a stable home environment - Many parents and children also described a strong and close parent-child relationship - When children felt emotionally secure, the children had better self-esteem and confidence | “She needs to know what will happen, not just when we visit the hospital, but also in general, so that she can feel secure” (Mother of a child aged 8-12 years) |
| **7. Growing up with BEEC** | | |
| **Subcategory** | **Content of subcategory** | **Quotes** |
| - 1. Future | - Children had thoughts, concerns and hopes for the future; worry for continence surgery, about career, parenthood, ability to travel | "Yes, I have kind of thought about the future, if you are kind of pregnant, will the umbilicus to stand out? Do you do a caesarean section, or how do you do it?" (Child 12-17 years with BEEC) |
| 7.2 Independence and Responsibility | - Younger children with BEEC often needed their parents to help them out, as children grew, they took more responsibility for bladder management and planning - Children with BEEC also had parents that could not completely let go of control in helping their child in the management of their condition | “Our son who leaks urine, brings incontinence products with him. He makes sure it is always ready and always with him.” (Mother of a child 12-17 years with BEEC) |
| 7.3 Intimacy and Sexuality/Sex | - Parents of children with BEEC rarely discussed questions about intimate relationships or sexuality with the child, but believed their children do think about this and several children had tried to ask their parents directly - Ability for a boy to get erection was important for HRQoL | “When my son has a lot of questions about sex, I usually say, bring it up with dad or the surgeon, because it's a bit difficult” as a mom (Mother of child 8-12 years with BEEC) |
| 7.4 When you get older and the body changes | - Entering puberty, adolescents with BEEC had bodily changes, some experienced less urinary leakage and were described to have typical teenage behaviors, including increased need to spend more time alone, being more introvert, and becoming more interested in finding a partner | "For me, I think, I kind of leaked less and less. Since I was in second grade, and then maybe in sixth grade or seventh grade, I quit using incontinence products" (Child 12-17 years with BEEC) |
| 7.5 Changes after continence surgery | - Children experienced the initial time after continence surgery as difficult - Children felt that the child’s continence surgery led to significant improvements with less urine leakage, reduced pain and fewer infections. It made it important to follow a strict schedule for bladder emptying. - Children felt that using Mitrofanoff was quick, could be carried out in different settings and made their daily lives easier | “There is usually a lot of snow in the winter. Well, that's a huge advantage because then you can manage clean intermittent catheterization trough Mitrofanoff to empty your bladder, you don't have to bring down your clothes all the way. It's so cold, but you can just pull up the zipper a little and you'll be fine. You don't get cold" (Child 12-17 years with BEEC) |
| BEEC, Bladder exstrophy-epispadias complex; CIC, Clean Intermittent Cathetherization; FG, focus group; HRQOL, health-related quality of life | | |
